# The role of the serotonergic and GABA system in translational approaches in drug discovery for anxiety disorders

**DOI:** 10.3389/fphar.2013.00074

**Published:** 2013-06-11

**Authors:** Jocelien D. A. Olivier, Christiaan H. Vinkers, Berend Olivier

**Affiliations:** ^1^Department of, Women’s and Children’s Health, Uppsala UniversityUppsala, Sweden; ^2^Center for Gender Medicine, Karolinska InstitutetStockholm, Sweden; ^3^Division of Pharmacology, Utrecht Institute for Pharmaceutical Sciences and Rudolf Magnus Institute of Neuroscience, Utrecht UniversityUtrecht, Netherlands; ^4^Department of Psychiatry, Rudolf Magnus Institute of Neuroscience, Utrecht Medical CenterUtrecht, Netherlands; ^5^Department of Psychiatry, Yale University School of MedicineNew Haven, CT, USA

**Keywords:** translational, animal model, GABA_A_, 5-HT, 5-HT_1A_ receptor, 5-HT_2_ receptor, 5-HTT, 5-HTTLPR

## Abstract

There is ample evidence that genetic factors play an important role in anxiety disorders. In support, human genome-wide association studies have implicated several novel candidate genes. However, illumination of such genetic factors involved in anxiety disorders has not resulted in novel drugs over the past decades. A complicating factor is the heterogeneous classification of anxiety disorders in the Diagnostic and Statistical Manual of Mental Disorders (DSM-IV-TR) and diverging operationalization of anxiety used in preclinical and clinical studies. Currently, there is an increasing focus on the gene × environment (G × E) interaction in anxiety as genes do not operate in isolation and environmental factors have been found to significantly contribute to the development of anxiety disorders in at-risk individuals. Nevertheless, extensive research on G × E mechanisms in anxiety has not resulted in major breakthroughs in drug discovery. Modification of individual genes in rodent models has enabled the specific study of anxiety in preclinical studies. In this context, two extensively studied neurotransmitters involved in anxiety are the gamma-aminobutyric acid (GABA) and 5-HT (5-hydroxytryptamine) system. In this review, we illustrate the complex interplay between genes and environment in anxiety processes by reviewing preclinical and clinical studies on the serotonin transporter (5-HTT), 5-HT_1A_ receptor, 5-HT_2_ receptor, and GABA_A_ receptor. Even though targets from the serotonin and GABA system have yielded drugs with known anxiolytic efficacy, the relation between the genetic background of these targets and anxiety symptoms and development of anxiety disorders is largely unknown. The aim of this review is to show the vast complexity of genetic and environmental factors in anxiety disorders. In light of the difficulty with which common genetic variants are identified in anxiety disorders, animal models with translational validity may aid in elucidating the neurobiological background of these genes and their possible role in anxiety. We argue that, in addition to human genetic studies, translational models are essential to map anxiety-related genes and to enhance our understanding of anxiety disorders in order to develop potentially novel treatment strategies.

## INTRODUCTION

Anxiety disorders constitute one of the most prevalent classes of psychiatric disorders. Anxiety disorders in the Diagnostic and Statistical Manual of Mental Disorders (DSM-IV) include panic disorder (PD), generalized anxiety disorder (GAD), phobias, social phobia, obsessive compulsive disorder (OCD), and post-traumatic stress disorder (PTSD) and were the most common mental disorders within Europe in 2010 with 14% prevalence (Wittchen, 2011). The heterogeneous classification system of individual anxiety disorders in the DSM-IV is based on symptomatology rather than etiology (Friedman et al., 2011). Anxiety disorders are often comorbid with other psychiatric disorders such as mood disorders and substance abuse disorders which make anxiety disorders in general a heterogeneous class of psychiatric disorders. Moreover, specific anxiety disorders are often incomparable to each other with regard to the symptomatology. Specifically, OCD and PTSD are quite different with regard to their symptomatology compared to GAD or social phobia. Application of the DSM-V has not resulted in major changes in the classification of anxiety disorders (Friedman et al., 2011). The complex and specific classification of anxiety disorders may eventually hinder drug development as one-to-one translation to preclinical models is not possible. A classification based on the neurobiological mechanisms underlying pathological anxiety (intermediate phenotypes) has been proposed as a novel strategy to discover novel targets to treat anxiety symptoms (Ressler and Mayberg, 2007). However, so far, progress in understanding the neurobiology of emotional (dys)regulation has not resulted in novel treatments. Another approach is based on the hypothesis of dysfunctional neurotransmitter systems, which assumes that anxiety disorders are associated with abnormal functionality of specific neurotransmitter systems. However, the definition of a “dysfunctional” neurotransmitter system is difficult and not without confounds, and even more, a direct and consistent relation between specific neurotransmitters systems and anxiety disorders has not been established. The difficulty of developing novel anxiolytic drugs is illustrated by the fact that existing anxiolytic drugs such as benzodiazepines (BZs) and selective serotonin (5-hydroxytryptamine, 5-HT) reuptake inhibitors (SSRIs) have been developed several decades ago. The development of BZs was the result of serendipity and SSRIs were primarily developed to treat major depressive disorder. Interestingly, when patients start using SSRIs they experience anxiogenic effects of the drug, while after administration of several weeks SSRIs work anxiolytically. This is probably due to activation of 5-HT_2C_ receptors as antagonists for these receptors are able to reverse the acute anxiogenic effect of SSRIs (Bagdy et al., 2001). Moreover, chronic exposure to SSRIs has been shown to downregulate 5-HT_2C_ receptors in the cortex (Attar-Levy et al., 1999). Although extensive search has occurred in the ensuing decades to find new targets for anxiolytics based on the known and anticipated neurochemical mechanisms underlying anxiety, no real breakthroughs have emerged. Currently, SSRIs remain the preferred drugs for the treatment of anxiety disorders (augmented with BZs for a limited time interval). Although efficacious, some patients are treatment resistant, and inherent disadvantages with regard to side effects are attached to the use of SSRIs and BZs. Therefore, preclinical and clinical studies over the last decades have focused on other mechanisms to target anxiety processes in order to ultimately treat anxiety disorders. Since then, evidence has emerged for several novel anxiety targets, including the corticotropin-releasing factor 1 (CRF_1_) receptors, neurokinin 1 (NK-1) receptors, and glucocorticoid receptors (for review, see Cryan and Sweeney, 2011). Although the preclinical efficacy of drugs targeting these neurotransmitters was often encouraging during development, no superior therapeutic anxiolytic effects have been found in subsequent clinical trials. Thus, no novel anxiolytic drug targets have reached the market to replace the current treatment choices. Surprisingly, even though targets such as the serotonin 1A (5-HT_1A_) receptor, serotonin 2 (5-HT_2_) receptor, the serotonin transporter (5-HTT), and the gamma-aminobutyric acid-A (GABA_A_) receptor have yielded drugs with known efficacy, the relation between the genetic background of these targets and anxiety symptoms and disorders is little understood. The complexity of anxiety disorders calls for a multidisciplinary approach of the phenomenon of anxiety. In this review, we argue that by using translational preclinical models, anxiety-related genes can be mapped and this information may subsequently be used to enhance our understanding of clinical studies in order to identify novel drug targets for anxiety. To this end, this review focuses on the convincing evidence stemming from preclinical and clinical studies that genes involved in the serotonin and GABA system play a pivotal role in the development of anxiety disorders. These 5-HTergic and GABAergic genes are highlighted throughout this review to illustrate the complexity of the genetic background of anxiety disorders.

## HERITABILITY OF ANXIETY DISORDERS

From family studies, the risk to develop an anxiety disorder such as PD, phobias, OCD, or GAD is three- to sixfold higher in first-degree relatives of affected probands compared to unaffected individuals (Chantarujikapong et al., 2001; Hettema et al., 2001). Also, twin studies have aided in estimating the genetic and environmental components of the variance in PTSD (Seedat et al., 2001). Moreover, concordance rates in monozygotic twins have been found to be increased compared to dizygotic twins for anxiety disorders with an estimated heritability in the range of 20–40% (Hettema et al., 2005). Thus, it is clear that genetic variability contributes to a certain extent to the risk for anxiety disorders. Nevertheless, the rather limited heritability implies that anxiety disorders are not mere genetically defined disorders.

### LINKAGE MAPPING

When a disease runs in the family, genetic markers in several generations can be studied, and such linkage mapping has been found to be critical for identifying genes that are causal for anxiety disorders. It is assumed that a gene contributing to a disease is located in the same area of the genome as the genetic marker. Significant linkage for anxiety and PD has been reported at chromosomes 9q31 (Thorgeirsson et al., 2003), 2q (Fyer et al., 2006), 7p (Knowles et al., 1998), 13q (Hamilton et al., 2003), 15q (Fyer et al., 2006), and 22q (Hamilton et al., 2003). Significant linkages were also found for a “PD syndrome” (13q; Weissman et al., 2000). Additionally, suggestive-linkage regions were reported for agoraphobia (1q; Gelernter et al., 2001), PD and agoraphobia (12q; Smoller et al., 2001a), social phobia (Gelernter et al., 2004), and markers for specific phobia on chromosome 14 (Gelernter et al., 2003). Finally, Kaabi et al. (2006) detected a strong linkage signal for anxiety and PD on chromosome 4 (4q31-q34) at marker *D4S413*. Linkage studies for OCD have only revealed single nucleotide polymorphisms (SNPs) in the glutamate transporter gene SLC1A1 on 9p24 (Walitza et al., 2010). Although linkage studies have implicated several chromosomal regions that may harbor susceptibility genes (Smoller et al., 2008a), no candidate genes have emerged that play a straightforward role in the expression for a vulnerability to anxiety or anxiety disorders (Smoller et al., 2009). This is probably due to the fact that several genetic risk factors only explain a limited amount of variance. Moreover, the strongest linkage signals seem to derive from recessive and highly penetrant diseases. Linkage studies are good to detect regions involved in these recessive diseases and can narrow down the search for causal variants to a few million base pairs.

### ASSOCIATION MAPPING

Association studies compare allelic variation in a group of patients to a healthy control population. When a certain allele plays a role in the development or susceptibility of a disease or is correlated with a causal allele, the frequency of this allele will be increased in the case population compared to the control population. Usually, linkage studies result in a signal of candidate genes in a certain region and are followed by a genetic association study on alleles in that genetic region. In this way, a specific gene or even a specific allele can be identified to play a causal role in a certain disorder. Genetic association studies have become the predominant method for identifying susceptibility loci for complex traits (Smoller et al., 2008a). Several genes have been associated with PD (Hovatta and Barlow, 2008), such as, e.g., catechol-*o*-methyl transferase (COMT; Hamilton et al., 2002; Domschke et al., 2004; Woo et al., 2004; Rothe et al., 2006), adenosine A2A receptor (ADORA2A; Deckert et al., 1998; Hamilton et al., 2004), cholecystokinin (CCK; Wang et al., 1998; Hattori et al., 2001; Maron et al., 2005), CCK-B receptor (Kennedy et al., 1999), 5-HT_1A_ receptor (HTR1A; Maron et al., 2004; Rothe et al., 2004; Fakra et al., 2009; Choi et al., 2010), 5-HT_2A_ receptor (HTR2A; Inada et al., 2003; Maron et al., 2005), 5-HTT (SLC6A4; Lesch et al., 1996; McDougle et al., 1998; Bengel et al., 1999; Ohara et al., 1999; Lee et al., 2005; Munafo et al., 2005), monoamine oxidase A (MAO-A; Deckert et al., 1999; Inada et al., 2003; Maron et al., 2004; Samochowiec et al., 2004), and the regulator of G protein signaling 2 (Rgs2; Smoller et al., 2008b). Genetic association studies have also revealed several genes involved in PTSD, with most attention for genes involved in the hypothalamus–pituitary–adrenal (HPA) axis and the regulation of neurobiological pathways such as the SLC6A4, DAT1, DRD2, DRD4, FKBP5, and GCCR (systematically reviewed in Digangi et al., 2013). Although linkage studies only found SNPs in the SLC1A1 to be associated with OCD, association studies have found more genes such as SLC6A4, HTR1D, HTR2A, HTR2C, DRD4, DRD2, SLC1a1, GRIN2B, GABBR1, COMT, MAO-A, TPH1, TPH2, BDNF (brain-derived neurotrophic factor), NTRK2, OLIG2, and MOG (reviewed in Walitza et al., 2010). For GAD the Gad2, Rgs2, and Ppargc1a (in anxiety-spectrum disorders) were found to be associated (Sokolowska and Hovatta, 2013). Moreover in general CDH2, ALAD, PSAP, EPB41L4A, DYNLL2, and PTGDS that were associated with anxiety disorders (Sokolowska and Hovatta, 2013). Among all these genes, the SLC6A4 (5-HTT) is one of the most widely investigated genes in relation to anxiety-related personality traits (Munafo et al., 2003). Most association studies of anxiety disorder have focused on candidate genes, which are suspected to play a role in a particular anxiety disorder. This can be based on earlier biological evidence (biological candidate) or because these genes are located within loci previously implicated via linkage studies (positional candidates). However, most likely more than one gene variant is involved in the regulation of distinct emotional responses, which together with the environmental influence will determine who will become affected. Studies in animal models of anxiety have provided evidence for the involvement of certain genes. The most intensively studied candidate genes are related to neurotransmitter systems implicated in the regulation of anxiety, to various neuropeptides, and to stress-related genes, and for that reason have functioned as targets to develop anxiolytic drugs. These targets include 5-HT, noradrenalin (NE), glutamate, dopamine, GABA, RGS2, and neuropeptides (CRF, neuropeptide Y, BDNF). To address the complexity of psychiatric disorders, two strategies have evolved; (1) going “big” and (2) going “deep” (Smoller et al., 2009).

### GENOME-WIDE ASSOCIATION STUDIES

First studies have looked for SNPs based on an unbiased survey of the entire genome (genome-wide association studies, GWAS). The main aim of this strategy is to increase the explained variance of genetic studies by increasing the number of genes and, subsequently, sample size. By selecting a reduced set of SNPs that adequately represents the genetic variation, the whole DNA can be investigated with DNA chips measuring up to millions of SNPs. Although statistically stringent demands are uphold, the GWAS approach has resulted in the elucidation of genes and genetic variants involved in complex diseases such as autism and bipolar disorder although at a disappointing level (Chen et al., 2010; Gershon et al., 2011). With regard to anxiety, several anxiety genes have been found (Hovatta and Barlow, 2008), yet replication studies are lacking. Currently, GWAS are still on-going to localize and identify putative risk genes for anxiety disorders. The reason why GWAS were relatively unsuccessful for anxiety disorders is not fully clear. Several causes have been proposed, to name a few: (1) small effect sizes (Manolio et al., 2009); (2) new analytical approaches are necessary to detect more locations in the genome (Lubke et al., 2012); (3) epistasis, only a few genes together could contribute to a genetic risk while a gene on its own will never be identified. Network- and pathway-based methods are necessary in identifying candidate genes and to provide functional links to connect genetic variants to phenotypes (reviewed in Sun, 2012); (4) copy number variations (CNVs) might be responsible for a non-trivial proportion of common risk disease. The majority of CNVs remain invisible to current GWAS technology and would require whole-genome sequencing instead; (5) epigenetic inheritance, all technologies that are used in GWAS are based on DNA sequence, however not all inherited information is carried in the DNA. Therefore, GWAS are not detecting epigenetic variations and epigenome-wide association studies should be performed to discover epigenetically inherited variations; (6) gene × environment (G × E) effects, as in many psychiatric disorders, the environment induces complex G × E interactions which are hard to pick up by GWAS technology.

### ENDOPHENOTYPES

Instead of going big, another strategy that has been increasingly applied is going “deep,” i.e., the use of endophenotypes or intermediate phenotypes. These familiar or heritable traits are assumed to underlie anxiety disorders and may result in more insight into neurobiological mechanisms compared to classically defined anxiety disorder. Endophenotypes are particularly relevant in anxiety disorders, as the neural circuitry and central pathways mediating anxiety are relatively well known, partly because of extensive animal models and knowledge derived from functional magnetic resonance imaging (fMRI) studies in humans. Those enable the study of the relationship between activities in particular brain areas and anxiety. The amygdala, a limbic area involved in emotional processing, shows enhanced activity in phobias and PTSD compared to healthy individuals (Etkin and Wager, 2007). Such (endo) phenotypes are important targets for genetic studies because the link between genetic variation and disorder risk is reflected more directly, as, e.g., became clear when specific candidate polymorphism were associated with such brain parameters. A polymorphism in the promoter of the 5-HTT gene has been frequently associated with amygdala reactivity (Hariri et al., 2002; Hariri and Holmes, 2006) implicating the S-allele (low transcriptional activity of the 5-HTT) in the increased amygdala reactivity toward external stimuli (Hariri, 2009). Anxiety and related stress responses are conserved in mammals at different levels. Therefore, similar genes in humans and rodents may regulate critical aspects of anxiety. While in humans it is difficult to control the genetic heterogeneity and environmental influences, animal models provide the possibility to identify novel candidate genes under controlled circumstances. In the section “preclinical genetic approaches to anxiety”, we describe some animal models of anxiety that made it possible to study *in vivo* genetic associations at a functional level.

### CONCLUSION

In conclusion, genetic studies aiming to unravel the neurobiological background of anxiety disorders have proven to be challenging. This is likely due to a complex and polygenic genetic background of anxiety disorders in which many genes influence the risk to develop anxiety disorders, each of them with a small effect. Moreover, epistatic processes, having the ability to mask the phenotype derived from other genes, are also very likely to be involved whereas environmental factors induce complex G × E interactions. The fact that different susceptibility genes segregate in different families possibly plays a role, making it extremely difficult to detect relatively small and diverse effects. Reported genes that have been associated with anxiety disorders have often been followed by non-replications. The risk of false positives is considerable and meta-analysis studies are needed to hint at a putative susceptibility gene or definitively reject it. Even if replications have been found, the number of negative studies often exceeds the number of positive studies (Smoller et al., 2009). These challenges have led to a generally critical perspective in the search of mental illness genes (Muglia, 2011; Klein et al., 2012). Moreover, Crow (2011) critically questions why only 1–2% of the 80–90% heritability of major psychiatric diseases can be attributed to genes identified by linkage and association. This suggests that many loci with small effects are involved for the heritability of anxiety-related personality traits (Shifman et al., 2008).

## PRECLINICAL GENETIC APPROACHES TO ANXIETY

Despite extensive research, human linkage and association studies have not led to major breakthroughs so far. Therefore, it is of great importance to use other approaches in studying the involvement of genes in anxiety disorders as well. Animal pathology resembles human pathology to a certain (but varying) degree (Fernando and Robbins, 2011) and has greatly enhanced our knowledge in the neurobiological mechanisms underlying anxiety. Animal models can be powerful in dissecting putative genes in anxiety and anxiety-associated traits (Flint and Shifman, 2008; Kas et al., 2011), which can be used in parallel to human genetic studies. Because genomic technology advances rapidly, linkage between targets and neuronal circuitry and genetic factors involved in anxiety disorders are becoming increasingly elucidated. Fundamental research aimed at these targets may contribute to unraveling novel insights in anxiety processes and consequently engender new opportunities for drug discovery. The future needs a strict translational approach; data found in human (anxiety) research including genetic and environmental factors, should be used to formulate scientific approaches in animals and vice versa. In animals, we have the opportunity to apply cell-specific inducible knock-outs or knock-ins. Moreover, new optogenetic technology enables selective manipulation of cellular mechanisms and circuit functions linked to the gene’s suggested function (Tye and Deisseroth, 2012). The 5-HT_1A_ receptor, the 5-HT_2_ receptor, the 5-HTT, and the GABA_A_ receptor complex belong to the most known and discussed targets in the field and will therefore be discussed below. Human and animal research continues to find new mechanisms around these targets and involvement of these targets in neural networks involved in anxiety modulation, opening new possibilities to apply in animal models and human psychopathology.

### ANIMAL MODELS OF ANXIETY

The development of predictive animal models and genetically modified rodents has aided to clarify the role of several pharmacological molecules in brain circuits relevant to anxiety processes, including normal and abnormal behavior. Many animal models for anxiety are based on the natural behavior patterns of rodents (Rodgers et al., 1997). These ethologically based behavioral models include “approach-avoidance” tasks (Cryan and Holmes, 2005) where animals are exposed to aversive environments such as an open field, elevated plus maze or light/dark box and avoid the aversive arena (center of open field, open arms of elevated plus maze, light arena in light/dark box). Besides these unconditioned procedures also conditioned procedures have been used to model anxiety disorders, including conflict procedures such as the Vogel water-lick conflict test (Vogel et al., 1971), defensive burying tests (de Boer and Koolhaas, 2003), the four-plate test (Boissier et al., 1968), and fear-potentiated startle (Brown et al., 1951). Next to these tests also other parameters have been developed to assess anxiety such as the use of radiotelemetry to assess physiological parameters (Bouwknecht et al., 2007), social interaction tests (File and Seth, 2003), predator stress (Blanchard and Blanchard, 1971), and stress-induced vocalizations (Sanchez, 2003). All these models assess behavior that is functionally related to human anxiety as they show good face and construct validity.

### THE 5-HT_1A_ RECEPTOR

The 5-HT_1A_ receptor has been implied in anxiety because 5-HT_1A_ receptor agonists exert anxiolytic activity in rodent models of anxiety (Olivier et al., 1999). Although clinically, development of new 5-HT_1A_ receptor agonists for anxiety disorders (e.g., ipsapirone, gepirone, tandospirone, flesinoxan) failed, the 5-HT_1A_ receptor has received considerable interest as a critical target implied in anxiety (Olivier et al., 1999; Holmes, 2008; Lanfumey et al., 2008; Akimova et al., 2009; Savitz et al., 2009). 5-HT_1A_ receptors are G protein-coupled inhibitory receptors expressed in 5-HTergic neurons as autoreceptors and in non-5-HTergic neurons as heteroreceptors. The somatodendritic 5-HT_1A_ autoreceptor controls 5-HTergic tone via feedback inhibition, although recently it was shown that not all 5-HT neurons express the somatodendritic 5-HT_1A_ autoreceptor mRNA (Kiyasova et al., 2013). It has been hypothesized that desensitized 5-HT_1A_ autoreceptors delay the onset of action of SSRIs that act by enhancing brain 5-HT levels (Gardier et al., 1996; Blier et al., 1998). 5-HT_1A_ receptors are quite abundantly, although restrictedly present in some brain areas. Autoreceptors are mainly, if not only, found in the dorsal and median raphé nuclei, whereas postsynaptic heteroreceptors are found in high densities in limbic regions (including hippocampus) and in the frontal medial prefrontal and entorhinal cortices.

#### Human data

Genetic and imaging studies in humans suggest that 5-HT_1A_ receptor density or regulation are associated with anxiety, but also with the response to antidepressants (Lesch and Gutknecht, 2004). An association was found between a C(-1019)G polymorphism (rs6295G/C) in the promoter region of the 5-HT_1A_ receptor gene (Htr1a) and mood-related variables, including amygdala reactivity (Fakra et al., 2009). The G-allele is associated with enhanced raphé (presynaptic) 5-HT_1A_ autoreceptor expression but reduced postsynaptic 5-HT_1A_ heteroreceptor expression (Le FrancÇois, 2008). How such changes contribute to an anxious phenotype is not known yet. More polymorphisms in the Htr1a gene exist, but it is not clear whether they influence anxiety (Drago et al., 2008).

#### Preclinical data

5-HT_1A_ receptors were found to modulate anxiety. All generated 5-HT_1A_ receptor knock-out (5-HT_1A_^-/-^) mice in several strains displayed enhanced anxiety (Heisler et al., 1998; Parks et al., 1998; Ramboz et al., 1998), although the anxious phenotype was dependent on the paradigm used (Pattij et al., 2001). Interestingly, the Swiss-Webster 5-HT_1A_^-^^/^^-^ mouse displayed a reduced sensitivity to the anxiolytic and sedative effects of diazepam, a non-α-subunit selective GABA_A_-positive allosteric modulator (Sibille et al., 2000; Olivier et al., 2001), indicating changes in some α-subunits of the GABA_A_–BZ receptor complex. However, this BZ insensitivity did not occur in other strains (Olivier et al., 2001; Pattij et al., 2002). Apparently, dysfunction of the GABA_A_–BZ system is not a prerequisite for the “anxiogenic” phenotype of the 5-HT_1A_^-^^/^^-^ mouse. The anxiogenic phenotype in the 5-HT_1A_^-^^/^^-^ mouse was not responding to SSRIs (Santarelli et al., 2003), although Guilloux et al. (2006) showed that 5-HT_1A_^-^^/^^-^ mice on a C57Bl/6 background did respond better to SSRIs compared to wildtypes. Moreover, it appeared that overexpression of the 5-HT_1A_ receptor reduced anxiety (Kusserow et al., 2004). Rescue experiments of forebrain 5-HT_1A_ receptors showed that postsynaptic 5-HT_1A_ receptors are critical in the development of the anxiogenic phenotype in the null mutations (Gross et al., 2002). In addition, transgenic developmental overexpression of 5-HT_1A_ receptors in the rostral brain was sufficient to restore normal anxiety levels. Pharmacological blockade of 5-HT_1A_ receptors in early development, but not in adulthood, appeared sufficient to enhance anxious behavior in wildtype mice (Lo Iacono and Gross, 2008; Vinkers et al., 2010a). Zanettini et al. (2010) found increased social anxiety in the 5-HT_1A_^-^^/^^-^ mice, however this was reversed by postnatal handling, indicating that neural circuits involved with social anxiety are susceptible to early-life experiences. The complex regulation of anxiety processes during development and adulthood illustrates the complexity of the neural substrate. Also the genetic regulation of anxiety and its pathology makes it clear that straightforward and simple relationships between the function of a certain receptor and anxiety are not very likely. Richardson-Jones et al. (2010) were able to manipulate the level of presynaptic 5-HT_1A_ autoreceptors during adulthood without a concomitant change in postsynaptic 5-HT_1A_ heteroreceptors. Mice with higher (1A-high) or lower (1A-low) autoreceptor levels were tested on their stress vulnerability and response to antidepressants. 1A-low mice showed enhanced 5-HT tone and still respond to an SSRI, whereas 1A-high mice had decreased 5-HT tone and were unresponsive to SSRIs. The authors suggest that 1A-lows reflect human C/C, whereas 1A-highs model G/G carriers of the Htr1a C(-1019)G polymorphism. Such genetic mouse models are extremely useful in studying the underlying processes emerging in anxiety disorders. In addition, Bortolozzi et al. (2012) showed that C-1A-siRNA can be used *in vivo* to selectively silence the 5-HT_1A_ autoreceptor resulting in reduced 5-HT_1A_-autoreceptor expression, but no alterations in postsynaptic 5-HT_1A_ receptors. Interestingly C-1A-siRNA increased the SSRI-induce elevation of extracellular 5-HT. Effects were seen after i.c.v. and intranasal C-1A-siRNA infusion which opens up new possible therapeutic applications. In conclusion, by genetic manipulation it was possible to study the exact role of specific 5-HT_1A_ receptors (e.g., presynaptic autoreceptors and postsynaptic heteroreceptors) in anxious behavior. Rodents can be manipulated in one specific gene, or even in a specific area. By doing so, the involvement of different 5-HT_1A_ receptors within anxiety disorders can be unraveled, and anxiolytic drugs can be developed for more specific targets (e.g., targeting only postsynaptical 5-HT_1A_ receptors in the rostral brain). Moreover, changes in other mechanisms in the brain in the modified mutant mouse can direct us to putative other and relevant targets and mechanisms involved in the complex anxiety phenotypes emerging. For example Pet1, a transcriptional factor of the ETS (E-twenty six) family, is important for determining the identity of 5-HT neurons in the raphe. Kiyasova et al. (2011) investigated 5-HT neurons in the Pet 1 knock-out mice and discovered a subset of 5-HT neurons that were either Pet1-dependent or Pet1-resistant, which resulted in different morphological features. Moreover, Pet1 knock-out mice showed reduced anxiety-like behavior in conflict-tests, but increased fear in aversive conditioning paradigms. Thus, Pet1 plays an important role in the acquisition and maintenance of 5-HT identity (Gaspar and Lillesaar, 2012; Andrade and Haj-Dahmane, 2013). These findings suggest that the differentiation of subpopulations of 5-HT neurons could also be a factor contributing to the development of anxiety disorders.

### 5-HT_2_ RECEPTORS

#### Clinical data

The 5-HT_2_ receptor subtypes are implicated in anxiety and in the mechanisms of related treatments (Quesseveur et al., 2012). 5-HT_2_ receptors couple to multiple cellular signaling pathways and are involved in several physiological brain functions (Leysen, 2004). For example, when SSRIs are combined with 5-HT_2C_ receptor antagonists this may result in greater efficacy in reducing anxiety symptoms and improving sleep (Garner et al., 2009). Agomelatine is a melatonergic receptor (M1 and M2) agonist, but also contains 5-HT_2C_ receptor antagonistic properties, and has anxiolytic properties in patients with GAD (Stein et al., 2008). However, the initial promise for 5-HT_2C_ antagonists such as deramciclane in GAD (Naukkarinen et al., 2005) has still to be confirmed consistently within large randomized placebo controlled studies.

#### Preclinical data

As in patients with GAD, agomelatine has been shown to relieve anxiety-like behavior in animals (Millan et al., 2005). Moreover, the 5-HT_2C_ receptor antagonist SB242084 increased the response of SSRIs in animal models (Cremers et al., 2004). Interestingly, Gomes and Nunes-de-Souza (2009) showed that stimulation of 5-HT_2A/2C_ receptors rather than stimulation of 5-HT_1A_ receptors in the periaqueductal gray matter (PAG) attenuate anxiety-like behaviors in mice previously exposed to the elevated plus maze. Moreover, in mice intra-PAG infusions of mCPP (meta-chlorophenylpiperazine), a 5-HT_2B/2C_ receptor agonist, attenuated anxiety-like behavior in the elevated plus maze which was blocked by the 5-HT_2A/2C_ receptor antagonist ketanserin (Nunes-de-Souza et al., 2008). Thus 5-HT_2A/2C_ receptors within the PAG play a key role in the regulation of anxiety-like behavior in mice. With respect to interactions of the 5-HT_2_ receptor it was shown that CRF sensitized the 5-HT_2_ receptor-mediated signaling through the CRF_1_ receptor. This resulted into increased anxiety-like behavior in mice (Magalhaes et al., 2010), indicating a functional interaction between CRF and 5-HT. Furthermore, etifoxine, a GABA_A_ receptor potentiator, dose-dependently increased the number of punished crossing in a four-plate test in mice (decreased anxiety). Interestingly this anti-punishment effect was blocked when a 5-HT_2A_ antagonist was administered (Bourin and Hascoet, 2010). In addition co-administration of the 5-HT_2A_ receptor agonist DOI together with a subthreshold dose of etifoxine induced an anti-punishment effect as well. Together these data indicate that the effect of etifoxine was modulated by 5-HT_2A_ ligands and that GABA and 5-HT can be co-released and act as co-transmitters in some regions of the central nervous system (CNS; Bourin and Hascoet, 2010). Several studies have suggested that 5-HT_2A_ receptors modulate learning and memory (Meneses, 2007a, b). As such, Zhang et al. (2013) found that stimulation of 5-HT_2A_ receptors with the agonist TCB-2 enhanced the extinction of cued fear memory in mice after trace and delay fear conditioning paradigms, while blockage with MDL 11,939 showed the opposite effect. With respect to G × E interaction it was shown that maternal separation increased adult anxiety behavior (Huot et al., 2001; Kalinichev et al., 2002). Interestingly, Benekareddy et al. (2011) have shown that blockade of the 5-HT_2_ receptor (ketanserin) during early postnatal life prevented the increased anxiety seen in animals exposed to maternal separation. Moreover, the enhanced 5-HT_2A_ receptor mRNA in the prefrontal cortex was also blocked by postnatal treatment of ketanserin, implicating that the 5-HT_2_ receptors are involved in the adverse effects of maternal separation. In addition to the pharmacological stimulation and blockage of the 5-HT_2_ receptor, disruption of the 5-HT_2A_ receptor in mice increased anxiety-like behavior in conflict anxiety paradigms as well (Weisstaub et al., 2006). Martin et al. (2013) created a mouse model expressing only the fully edited VGV isoform of the 5-HT_2C_ receptor and showed that these mice had increased anxiety-like behavior after stimulation with a 5-HT_2C_ receptor agonist in the social interaction test. Moreover, in response to an innately aversive ultrasonic stimulus these mice freezed significantly more and displayed decreased brain 5-HT turnover during stress. When these results were put in relation with the 5-HT_2C_ receptor mRNA splicing process it turned out that the truncated protein (5-HT_2C_ receptor-Tr) interacted with the full-length receptor (5-HT_2C_ receptor-Fl). The 5-HT_2C_ receptor-Tr was localized in the endoplasmic reticulum where it bound to the 5-HT_2C_ receptor-Fl. As a result, the 5-HT_2C_ receptor-Fl could not reach the plasma membrane (Martin et al., 2013). These results show that the 5-HT_2C_ receptor pre-mRNA editing and splicing altering 5-HT_2C_ receptor levels are involved in pathological conditions. Finally, the decreased sociability and sniffing induced by mCPP (5-HT_2B/2C_ agonist) in 5-HTT^+^^/^^+^ mice, was not seen in 5-HTT^-^^/^^-^ mice (Moya et al., 2011) which is probably due to increases in RNA editing of the 5-HT_2C_ receptor in the amygdala of 5-HTT^-^^/^^-^ mice that generates less active reporter isoforms. In conclusion, the 5-HT_2_ receptors are involved in anxiety processes; however more research is needed to further dissect the physiological relevance in different brain regions.

### THE SEROTONIN TRANSPORTER (5-HTT)

#### Human data

The 5-HTT has been implied in processes underlying mood, anxiety and associate disorders mainly because SSRI anxiolytics block 5-HT uptake into the neuron thereby increasing 5-HTergic output. Polymorphisms in the promoter of the 5-HTT gene (5-HTTPRL) and its associated transcriptional control region, influence the functioning of the 5-HTergic system (Lesch, 2001). Variable numbers of tandem repeat polymorphisms are known in intron 2 as well as several SNPs that influence the structure of the 5-HTT protein (Murphy and Lesch, 2008). This makes the modulation of 5-HTergic transmission via the 5-HTT mechanism highly complex and gives probably an important insight in the factors that play a role in the genetic complexity of any psychiatric disorder. Gene variations influence intermediate biological phenotypes in concert with other genes, epigenetic variation, environmental and developmental factors. All these complex interactions contribute to the risk or resilience to develop a psychiatric condition. One avenue to pursue would be to try to find associations between specific candidate genes and intermediate phenotypes mediating between a moderating allele and a more complex disease phenotype (Murrough and Charney, 2011).

The 5-HTTLPR allele variations are called the short “S” allele and the long “L” allele. The S-allele has 44 base pairs less and lower transcriptional activity of the 5-HTT gene than the L-form. The S-allele has been the focus of many association studies (Hamilton, 2009). Although negative studies and non-replications with anxiety phenotypes have been reported (e.g., Risch et al., 2009; Grabe et al., 2011), many reported associations with anxiety-related traits and anxiety disorders (Lesch et al., 1996; McDougle et al., 1998; Bengel et al., 1999; Ohara et al., 1999; van Gestel et al., 2002; Lee et al., 2005; Munafo et al., 2005). Considerable evidence showed that after stressful life-events the low expression S-allele is associated with *poorer* outcomes (Lesch et al., 1996; Caspi et al., 2003). A significant interaction between maternal anxiety during gestation and subsequent levels of infant negative emotionality at 6 months of age was modulated by the 5-HTTLPR of the child (Pluess et al., 2011). Moreover, SS-allele carriers appeared particularly sensitive toward unpredictability as seen by modulated attention to the stress (Drabant et al., 2012), suggesting that such a mechanism may underlie the risk for psychopathology. In addition, deductive reasoning appeared also dependent on 5-HTTPRL genotype. Differences in 5-HTT functioning renders some individuals more vulnerable to emotional factors, thereby generating a deleterious effect on rational reasoning (Stollstorff et al., 2013). A gene × gene interaction was found between the 5-HTTLPR (measure in LL-variants) and an oxytocin receptor variant (TT variant of the SNP rs2268498) on individual differences in negative emotionality (Montag et al., 2011). Such data indicate that 5-HTergic and oxytocinergic neurotransmission processes are somewhere entwined and seem to play a role in affective disorders. In general, S-alleles of the 5-HTTLPR are associated with increased risk for a variety of psychiatric disorders, including anxiety. Thus, the S-allele is considered a “risk” or “vulnerability” allele (Caspi et al., 2010) whereas the function of the L-allele is far less clear although this allele has been suggested as a potential risk factor for the development of psychopathic traits too (Glenn, 2011). Because every human has either L, S or both alleles and most people do not suffer from psychiatric abnormalities; it must be assumed that the genome includes several “protective” alleles that make many individuals resilient to stress and pathology. Such protective genes have been suggested, e.g., the CRF_1_-receptor variants that have been associated with protection from the extreme stresses of maltreatment during childhood (Polanczyk et al., 2009) and protective, emotional-resilience enhancing effects of the L-allele in students (Stein et al., 2009). Belsky et al. (2009) suggested that S-allele carriers are more vulnerable in general, not only negatively, but also positively. Thus “vulnerability genes” or “risk alleles” seem to make individuals more susceptible to environmental influences, for better and for worse. Homberg and Lesch (2011) take the hypothesis that S-carriers perform better in cognitive tasks than L-carriers and argue for a switch from a deficit-orientated connotation of the 5-HTTLPR variants to a cognitive superiority of S-allele carriers (which have enhanced reactivity of corticolimbic neural circuitry). Environmental conditions will determine whether a positive (cognitive) or negative (emotional) response will happen. Also Hankin et al. (2011) showed that SS-allele children were more sensitive to the environment. Under unsupportive, non-positive parenting SS-allele children exhibit low levels of positive affect, but with supportive/positive parenting these children displayed higher levels of positive affect. In addition to this, Eley et al. (2012) showed an association between the 5-HTTLPR and response to psychological treatment. That is, SS-allele children with anxiety disorder respond up to 20% better to psychotherapy compared to L-allele (SL/LL) carriers. This environmental sensitivity of the 5-HTTLPR makes it even more difficult, and should be taken into account when treating anxiety disorders.

#### Preclinical data

Several animal models were created to study the role of the 5-HTT and altered 5-HT signaling in the *in vivo* actions of SSRIs. For instance, Thompson et al. (2011) created a knock-in mouse expressing 5-HTT M172, which did not affect the recognition of 5-HT, but affected the serotonergic system and emotional behavior. Also, mice overexpressing 5-HTT have been generated resulting in reduced anxiety levels and bodyweight (Jennings et al., 2006; Line et al., 2011) and enhanced 5-HT_2A/C_ receptor function (Dawson et al., 2011). This model, together with 5-HTT knock-out (5-HTT^-^^/^^-^) models might eliminate the effects of lifelong 5-HTT disturbances with all the compensatory effects occurring over the life span. Both 5-HTT^-^^/^^-^ mouse (Bengel et al., 1998) and rat (Smits et al., 2006) have been created. 5-HTT^-^^/^^-^ rodents display increased extracellular 5-HT in several brain regions (Fabre et al., 2000; Mathews et al., 2004; Shen et al., 2004; Homberg et al., 2007a; Olivier et al., 2008). Due to these increased extracellular 5-HT levels alterations in neurodevelopment and 5-HT synthesis/metabolism are found (reviewed in Murphy and Lesch, 2008; Homberg et al., 2010). 5-HTT^-^^/^^-^ rodents have been considered as an extreme model of the 5-HTTLPR polymorphisms in humans, as brain and behavioral phenotypes of 5-HTT^-^^/^^-^ animals resemble the heterogeneity observed for the 5-HTTLPR (Hariri and Holmes, 2006; Wellman et al., 2007; Homberg et al., 2008b; Olivier et al., 2008).5-HTT^-^^/^^-^ animals have an altered ability to cope with stress and display anxiogenic and depressogenic behavior (Holmes et al., 2002; Tjurmina et al., 2002; Adamec et al., 2006; Wellman et al., 2007; Olivier et al., 2008; Jansen et al., 2010; Kalueff et al., 2010). Interestingly, when the environment is rewarding, 5-HTT^-^^/^^-^ rodents are more hypersensitive as shown by their increased sensitivity for psychostimulants (Sora et al., 1998, 2001; Homberg et al., 2008a; Nonkes et al., 2013) indicating that 5-HTT^-^^/^^-^ rodents are more sensitive to the environment. As found in S-allele carriers (Roiser et al., 2006a, b, 2007; Finger et al., 2007), improved cognition has been observed in 5-HTT^-^^/^^-^ rodents (Homberg et al., 2007b, 2008b; Brigman et al., 2010) together with improved behavioral flexibility, directing their behavior toward the most rewarding stimuli (Brigman et al., 2010; Nonkes et al., 2013). Reduced conditioned freezing to a predicted foot shock is found in 5-HTT^-^^/^^-^ rodents when a positive stimulus was given (Nonkes et al., 2012). However, it appears that phenotypical plasticity is not only present in 5-HTT^-^^/^^-^ animals early in life, but also later in life (Homberg and van den Hove, 2012). This also accounts for heterozygous (5-HTT^+^^/^^-^) rodents, which might be considered as a more valuable model for the 5-HTTLPR model as they have reduced expression of the 5-HTT, comparable to the S-allele carriers. For instance, low maternal care increased anxiety-like behavior in adult 5-HTT^+^^/^^-^ mice, but not in wildtype littermates (Carola et al., 2008). This increased emotionality was linked to increased BDNF mRNA levels in the hippocampus, suggesting a role for BDNF in programing the 5-HTT^+^^/^^-^ brain to become more susceptible to the environment. Interestingly, only 5-HTT^+^^/^^-^ mice that experienced high maternal care showed increased 5-HT and norepinephrine levels in the hippocampus, together with decreased 5-HT turnover (Carola et al., 2011). At baseline level, 5-HTT^+^^/^^-^ mice display decreased emotional behavior, however, upon prenatal maternal restraint stress, 5-HTT^+^^/^^-^ offspring displayed increased emotional behavior (Van den Hove et al., 2011), although also decreased anxiety levels and enhanced memory performance were found in these mice. This is an important finding as individuals with anxiety symptoms have a range of biases in emotion processing, such as a willingness to selectively attend to thread cues (Bar-Haim et al., 2007; Waters et al., 2008) and to interpret emotionally ambiguous stimuli in a negative manner (Mathews and MacLeod, 2005). When 5-HTT^-^^/^^-^ and 5-HTT^+^^/^^-^ mice underwent a loser experience in a social defeat test they displayed delayed fear extinction and decreased recall of extinction to a higher extent than wildtypes (Narayanan et al., 2011). In addition, 5-HTT^-^^/^^-^ losers displayed increased anxiety levels and reduced exploration (Jansen et al., 2010). Similarly, increased escape latencies were found in 5-HTT^-^^/^^-^ and 5-HTT^+^^/^^-^ mice after repeated inescapable footshock stress (Muller et al., 2011). Moreover, chronic psychosocial stress due to an intruder in the cage resulted into decreased locomotor activity and increased social avoidance (Bartolomucci et al., 2010). It is clear that both the immature developing brain as well as the mature brain is sensitive to changes in the environment.

The advantage of having animal models for human disorders is that underlying mechanisms in the brain can be more easily studied as environmental influences can be regulated. With use of for example fMRI or microPET (micro-positron emission tomography) scanning, brain areas can be studied in humans. By doing so, it was discovered that the amygdala and prefrontal cortex of S-allele carriers showed hyperactivity upon environmental stimuli (Hariri et al., 2002; Kalin et al., 2008). However, a molecular understanding of this phenomenon is lacking, while such understanding might be helpful in identifying new targets for the diagnoses and therapy of anxiety disorders. With use of animal models it is possible to study the gene, the environment and their interactions. For example, low maternal care caused deficient GABA_A_ receptor binding in the amygdala during adulthood. In pups with low maternal care increased α-amino-3-hydroxy-5-methyl-4-isoxazolepropionic acid receptor binding was found in the hippocampus, which correlated with BDNF mRNA levels in the somatosensory cortex. These effects are independent of genotype, and are only environmental. However, lower maternal care in 5-HTT^-^^/^^-^ mice elevated BDNF mRNA levels in the hippocampus. Moreover, it was shown that loser stress in a resident-intruder test increased pronounced neuroplastic changes of pyramidal neurons in the prelimbic cortex and amygdala (Nietzer et al., 2011). Also effects on the corticolimbic system were found (reviewed in Homberg and van den Hove, 2012). While we now only discussed the G × E interactions, epigenetics is probably also a key contributor for these interactions. The relationship between genetic and epigenetic variation at the 5-HTT gene has so far not been studied in 5-HTT-deficient animal models. But 5-HTT-deficient rodents may be particularly suitable to study these interactions.

### GABA_A_ RECEPTOR α SUBUNITS AND ANXIETY

In the 1950s, BZs were serendipitously found as having therapeutically interesting activity with anxiolysis, sedation, anticonvulsive activity, and muscle relaxation. The molecular target of BZs is the GABA_A_ receptor (Möhler and Okada, 1977). BZs mediate their actions via a modulatory binding site that is present on most, but not all GABA_A_ receptors. The binding site for BZs is formed by one of the α subunits (α1, α2, α3, or α5) and a γ subunit (almost exclusively the γ2 subunit) GABA_A_ receptors are the main inhibitory neurons in the CNS and it is estimated that 20–30% of all neurons in the CNS are of the GABA_A_ type. BZs do not open the Cl^-^ channel in the absence of GABA. Only if the GABA receptor site is activated, activation of the BZ site may modulate the opening of the channel. Ligands at the BZ binding site are allosteric modulators. They modify the efficacy and/or affinity of GABA in positive (positive allosteric modulation, PAM), negative (negative allosteric modulation, NAM), or have neutral effects by stabilizing different three-dimensional conformations of the complex. Selectivity of a ligand for a specific receptor subtype can be obtained by affinity and/or by efficacy changes that determine the potential potency of a ligand.

#### Human data

Even though specific SNPs of the GABAergic system have been found to play a role in anxiety disorders (Nemeroff, 2003; Kalueff and Nutt, 2007), GWAS on anxiety disorders are scarce and GABAergic candidate genes emerging from the existing studies have been equivocal (Logue et al., 2012; Otowa et al., 2012). A limited number of studies suggested some link between the GABRA2 gene and anxiety. Nelson et al. (2009) found that polymorphisms in the GABRA2 gene interact with early childhood trauma and increase the risk for PTSD. Pham et al. (2009) found, investigating 26 SNPs in four GABA_A_ receptor genes (GABRA2,3,6 and GABRG2) that none of the allelic variation in these genes was involved in liability to anxiety-spectrum disorders. Besides the GABRA subunit genes, several other GABAergic systems have been implicated in the genetic load of GABA system pathways on the psychobiology of anxiety. Suggestive signals for an association with anxiety disorders and anxiety-related personality traits have been found for other genes, e.g., glutamic acid decarboxylase 1 (Hettema et al., 2006) and 2 (Smoller et al., 2001b), b3 subunits of the GABAA receptor (Feusner et al., 2001), the diazepam binding inhibitor (Thoeringer et al., 2007), and the GABA transporter 1 (Thoeringer et al., 2009). The latter authors suggest a multiple system hit-theory in the genetic basis of anxiety disorders; many loci at different genes of the GABA system, each with a small effect, contribute to an individual’s risk on anxiety disorder. If several risk genes are present, anxiety might develop depending upon adverse environmental (stress) factors.

#### Preclinical data

By making the GABA α subunits insensitive to the diazepam binding [α1(H101R) mice] strong evidence was gathered that α1 subunits were involved in sedative and anterograde amnesia effects of diazepam. As such α2 point mutations [α2(H101R) mice] led to absence of the anxiolytic and diminished muscle relaxant action, but intact anxiolysis. Point mutations in α3 [α3(H126R) mice] and α5 [α5(105R) mice] did not diazepam-induced myorelaxation, whereas sedation and anxiolysis were intact. Such data strongly suggest a functional differentiation in the GABA_A_ receptors depending on the α-subunit composition (for review, see Möhler, 2006; Rudolph and Knoflach, 2011). Classic BZs are still frequently prescribed, have therapeutic activity but, inherent to the activation of all relevant α-subunits, come with build-in side effects. If used as anxiolytic tool, sedation is one of the troubling side effects. Furthermore, upon chronic use, BZs can lead to dependency, tolerance and induce abuse liability limiting long-term use (Tan et al., 2011). Recent efforts have tried to synthesize new drugs that have selectivity and potency for specific α subunits (Rudolph and Knoflach, 2011) although relatively selective drugs for the α1 subunit are already in use for sedation/hypnotic purposes (zolpidem, zopiclone, (S)-zopiclone, and zaleplon). Compounds that selectively activate the α2 subunits and have no effects on any other α subunit might constitute an ideal, non-sedative anxiolytic, although activation of α3 subunits might contribute to an anxiolytic profile (Dias et al., 2005; Vinkers et al., 2009; Atack, 2010). L-838417, a partial PAM at α2, α3, and α5 containing GABA_A_ receptors and an antagonist at α1 containing receptors has a non-sedating anxiolytic profile in mice (McKernan et al., 2000; van Bogaert et al., 2006) and primates (Rowlett et al., 2005). Development of this compound has been stopped due to an unfavorable pharmacokinetic profile (Scott-Stevens et al., 2005). TPA023, an α2/α3 PAM, has anxiolytic and no sedative effects in rodents (Atack et al., 2006). TPA023 was evaluated in three phase 2 studies in GAD and showed preliminary indications of anxiolytic activity without sedation (Atack, 2010). However, this compound had to be withdrawn due to severe preclinical toxicity. A comparable story holds for ocinaplon, having a non-sedative anxiolytic profile in humans but also had to be withdrawn due to hepatotoxicity (Lippa et al., 2005; Czobor et al., 2010). Several other ligands have been synthetized and tested, mostly restricted to preclinical phases. It appears possible to make compounds with some selectivity for specific α subunits but *in vivo* efficacy is extremely difficult to design: both positive and negative allosteric modulators have been found, sometimes even mixed PAM/NAM effects on different α subunits are present or no selectivity is present *in vitro* whereas *in vivo* some efficacy is found (e.g., ocinaplon). MRK-409, an extremely low partial agonist (PAM) at α1, α2, and α5 containing GABA_A_ receptors but higher intrinsic activity at α3 subunit GABA_A_ receptors, appeared to be anxioselective in animals but sedative in humans, already at low (<10%) receptor occupancy (Atack et al., 2011). One of the unresolved issues around subunit selective GABAergic compounds is the issue of tolerance and abuse potential. Does activation of all α subunit containing GABA_A_ receptors lead to addiction or is that caused by specific α subunits? This is an important issue because the development of potentially abusive medications will meet severe constraints if not impossible. There is some evidence that activation of α1 subunits is essential in the addictive properties of BZs (Tan et al., 2010, 2011). However, the processes of tolerance development are complex and endpoint-dependent (Vinkers et al., 2012). If the therapeutic effects of activation of α1-containing GABA_A_ receptors cannot be separated from potential addictive side effects, no further development of α1 subunit specific ligands can be expected. However, if addictive properties are not entwined in (chronic) activation of the other α(2,3,5) subunits, new developments in the field of anxiety (and others like cognition and analgesia) might be expected (Mirza and Munro, 2010; Vinkers et al., 2010a).

### THE INTERACTION BETWEEN 5-HT AND GABA

The GABA and the serotonergic system may directly interact (Lista et al., 1989; Gao et al., 1993; Fernandez-Guasti and Lopez-Rubalcava, 1998). However, the evidence is equivocal (Shephard et al., 1982; Thiebot, 1986). A serotonergic component in the anxiolytic actions of GABAergic BZs has been suggested (Stein et al., 1977; Thiebot et al., 1984; Harandi et al., 1987). Moreover, studies have found that a decreased serotonin activity and turnover emerges after the administration of BZs (Chase et al., 1970; Stein et al., 1977; Pratt et al., 1979; Trulson et al., 1982; Wright et al., 1992), although others have not found such effects (Shephard and Broadhurst, 1982; Thiebot et al., 1984; Thiebot, 1986). Also, the vast majority of serotonergic neurons express GABA_A_ receptor α_3_-subunit immunoreactivity but not GABA_A_ receptor α_1_-subunit staining (Gao et al., 1993). This is remarkable as the α_1_ subunit is highly prevalent in the CNS. Thus, BZs could at least partially produce their anxiolytic effects by activating α_3_ subunits located on serotonergic neurons (Vinkers et al., 2010b). In support, serotonergic raphe nuclei receive a prominent GABAergic input via distant sources as well as interneurons (Harandi et al., 1987; Bagdy et al., 2000; Gervasoni et al., 2000; Varga et al., 2001; Vinkers et al., 2010b). Together, the interaction of the GABA and serotonin system in anxiety disorders could be valuable in the search for novel anxiolytic drugs. Nevertheless, the fact that BZs acutely reduce anxiety, whereas SSRIs take several weeks before anxiolytic activity becomes apparent suggests that the two drug classes exert their effects via different mechanisms.

## TRANSLATIONAL STUDIES INTO ANXIETY

Can the data on the involvement of 5-HT in anxiety and anxiety disorders (here illustrated with the 5-HTT, the 5-HT_2_ receptor and the 5-HT_1A_ receptor) be used to design translational research that possibly will generate new hypotheses and targets for anxiolytic therapeutics? Recently, Jasinska et al. (2012) formulated a hypothesis around the involvement of the 5-HTT gene, stress and raphe–raphe interactions in order to try to explain the risk of depression as a result of G × E interactions between the 5-HTT gene and stress. Different populations of 5-HTergic neurons in the dorsal raphe (DR) nucleus exist that differentially contribute to the response to stress. As mentioned before differentiation of subpopulations of 5-HT neurons could also be a factor contributing to the development of anxiety disorders (Gaspar and Lillesaar, 2012; Andrade and Haj-Dahmane, 2013). Although Jasinska et al. (2012) hypothesize this mechanism mainly for depression, there is no a priory reason why anxiety disorders would not be mediated by this or a similar mechanism. The authors propose that the variability in the reuptake of 5-HT during stressor-induced raphe–raphe interactions alters the balance in amygdala–ventromedial prefrontal cortex–DR (VMPFC–DR) circuitry. This VMPFC-DR circuitry is important in the reactivity to stressors and the regulation of emotion. In LL-individuals with an efficient 5-HT transport the circuitry is able to normalize, but not so in SS-individuals, potentially leading to abnormal activity and pathology. Whether such a mechanism also acts in human pathology is as yet unresolved but could lead to specific searches for new mechanisms causing pathological anxiety. Next to different functional 5-HTergic populations in the DR, 5-HTTs appear very dynamically regulated (Steiner et al., 2008), undergo regulated membrane trafficking as well as transitions between low and high activity states, with many signaling pathways involved. Moreover, 5-HTT exhibits dynamic associations with cytoskeletal binding proteins; actually Chang et al. (2012) found two pools of 5-HTT proteins on the surface of 5-HTergic cells, one relatively with free diffusion, the other with restricted mobility due to binding to the cytoskeleton. Whether the 5-HTergic system exerts this kind of extremely variability which might lead to new and better understanding of the role of the 5-HTT complex, including its genetic variability is still a matter of the future but it remains fully possible that new mechanisms involved in anxiety and its disorders might emerge.

## CONCLUDING REMARKS

This review has illustrated the complexity of research on the genetic background of anxiety disorders. Although we discuss only the serotonergic and the GABA system, more systems/candidates are of potential interest including glutamate, NE, dopamine, and some peptides (reviewed in Christmas et al., 2008), as well as specific translocator protein which promote neurosteroidogenesis (Taliani et al., 2009; Nothdurfter et al., 2012) and agomelatine (Stein et al., 2008). However, in the present review, four targets have been presented to exemplify the complexity of anxiety: the 5-HT_1A_ receptor, 5-HT_2_ receptor, 5-HTT, and GABA_A_ receptor. This is important as two known class of drugs (SSRIs and BZs) are effective anxiolytics. Even though these anxiolytic drugs have been around for decades, no subsequent breakthrough has become available. The reasons for the relative lack of progress in the anxiety field are not completely clear but may be due to the heterogeneous classification of anxiety disorders, but also the complex regulatory and financial regulations in the finding of new “druggable” targets (beyond the scope of this review, see, e.g., Knutsen, 2011). Nevertheless, a recurring theme is the continued paucity of novel targets for anxiolytic drugs and our limited knowledge of the mechanisms underlying the various anxiety disorders. This includes the limited contribution of genetic studies to novel anxiolytic targets. In this review, we have argued that it is vital to invest in fundamental research in the mechanisms involved in anxiety processes in animals and unaffected individuals. Because a direct investigation of the human brain is often not possible, animal research may contribute considerably in finding neural substrates for anxiety and its pathology. However, it is not realistic to think that such knowledge is completely translatable to the clinical situation. Moreover, in animal models it is not always possible to model specific symptoms related to human pathology, which might cause limitations in the development of novel drug targets.

The initial hope was, after elucidation of the human genome, that the identification of causative genes would be a matter of time. Notwithstanding a certain degree of heritability of anxiety disorders, no single gene or set of genes has emerged from a large number of studies on large cohorts of patients thus far. It becomes increasingly evident that anxiety disorders, probably similar to the neurobiological mechanisms underlying anxiety processes, are the result of many hundreds of genes with small effects which display complex interactions with both environmental factors and other genes. Therefore, genetic approaches in studies on anxiety disorders may be enriched with preclinical studies to identify relevant drug targets. It is improbable that a single gene contributes significantly to anxiety processes to a large degree. It is striking that the functionality of GABA and 5-HT system in “normal” or “pathological” anxiety in healthy individuals is largely unknown, In case of 5-HT modulation (via 5-HT_1A_ receptor activation or blockade of the 5-HTT) an indirect effect is possibly the most logical explanation, because treatment of anxiety disorders with SSRIs or buspirone takes weeks or even months before anxiolytic activity is seen (acute effects seen after administration of these drugs are even anxiogenic). The delayed effect therefore points to induction of mechanisms that slowly change and need time to become effective (plasticity changes). Anxiolytic effects after activation of GABA_A_ receptors seem acute and might point to a primary mechanism directly involved in anxiety regulating mechanisms. Close collaboration between fundamental research and clinical studies into the mechanisms underlying anxiety might lead to breakthroughs in the search for novel anxiolytic drugs and enhance the success of research and development efforts aimed at drug discovery for anxiety disorders. In conclusion, we argue that animal models should play an important role in the future anxiolytic drug development as a fundamental component of a broad multidisciplinary approach. To be successful, novel clinical insights into the etiology of anxiety disorders from preclinical studies must be integrated in the broader context of human genetic studies and novel biopathway analysis.

## Conflict of Interest Statement

The authors declare that the research was conducted in the absence of any commercial or financial relationships that could be construed as a potential conflict of interest.
